# Emergence of a novel GII.17 variant is associated with immune evasion rather than an altered HBGA-binding profile

**DOI:** 10.1371/journal.ppat.1014522

**Published:** 2026-08-21

**Authors:** Stefan T. van der Krieken, Francisco Morais Esteves, Nele Villabruna, Claudia M. E. Schapendonk, Ray W. Izquierdo-Lara, Putri Ayu Fajar, Lonneke Leijten, Anja de Jong, Roman I. Koning, Michail C. Doukas, Corine H. GeurtsvanKessel, Marion P. G. Koopmans, Miranda de Graaf

**Affiliations:** 1 Department of Viroscience, Erasmus Medical Center, Rotterdam, Netherlands; 2 Institute of Biochemistry and Research Center for Emerging Infections and Zoonoses (RIZ), University of Veterinary Medicine Hannover, Hannover, Germany; 3 Department of Pathology, University of Veterinary Medicine Hannover, Hannover, Germany; 4 Electron Microscopy Facility, Cell and Chemical Biology, Leiden University Medical Center, Leiden, Netherlands; 5 Department of Pathology, Erasmus MC Cancer Institute, Erasmus Medical Center, Rotterdam, Netherlands; US Food and Drug Administration, UNITED STATES OF AMERICA

## Abstract

The epidemiology of noroviruses, a major cause of gastroenteritis in humans, is shaped by their continuous evolution. Novel genotypes and variants periodically emerge, replacing previously dominant strains. In 2024, a novel GII.17 variant led to an increased number of notified outbreaks, becoming the dominant genotype detected in Europe. To investigate if this surge in GII.17 detection is attributable to changes in the binding and antigenicity of this novel variant, we compared properties of a GII.17 2024 strain with representative GII.17 strains from 2005 (Clade B), 2014 (Clade C), and 2015 (Clade D), and the epidemic GII.4 Sydney 2012 variant. Norovirus virus-like particles (VLPs) were used to assess binding to saliva samples and human intestinal tissues with different histo-blood group antigen (HBGA) profiles. Binding specificity to individual glycans was determined using synthetic HBGAs. Additionally, VP1-NanoLuc (NLuc) fusion proteins were used in binding blocking assays to evaluate the antigenic properties of the variants using 75 sera from healthy adults that were collected during different time periods of GII.17 circulation (the winters of 2009–2010, 2015 and 2024). Compared with other GII.17 variants, the 2024 strain exhibited amino acid substitutions in the P2 domain, which contains both antigenic sites and the HBGA binding site. Binding-blocking assays with human sera confirmed that the 2024 variant is antigenically distinct from the 2005 and 2015 GII.17 strains but closely related to the 2014 variant. All post-2005 GII.17 strains showed a broader HBGA binding profile compared to the GII.17 2005 strain, likely contributing to their more successful spread. These findings show that the recent re-emergence of GII.17 coincided with antigenic drift, enabling it to escape from pre-existing immunity, but not with major changes in HBGA binding specificity. Understanding the factors influencing norovirus emergence is essential for the development of preventive strategies against future norovirus epidemics.

## Author summary

Noroviruses are a leading cause of acute gastroenteritis worldwide and are responsible for large numbers of outbreaks each year. New norovirus variants can spread rapidly when they are less well recognized by pre-existing immune responses in the population. In 2024, a new GII.17 variant became increasingly common in several regions, raising questions about why this virus was able to spread so successfully. In this study, we compared this new variant with other GII.17 viruses that circulated in 2005, 2014, and 2015. We examined how well these viruses could attach to specific sugars and tissues in the human intestine and how well they were recognized by antibodies in human serum samples collected over time. We found that the 2024 variant was less well recognized by pre-existing antibodies than some earlier strains, suggesting that it had changed in ways that may help it evade prior immunity. In contrast, its ability to bind to sugars and intestinal tissues was broadly similar to that of other recent variants. Together, our findings suggest that changes in immune recognition, rather than major changes in binding specificity, contributed to the recent emergence of this GII.17 variant.

## Introduction

Human noroviruses are the principal etiological agents of viral gastroenteritis and are transmitted via the fecal-oral route. It is estimated that norovirus is responsible for 677 million cases of acute gastroenteritis and more than 200,000 deaths globally each year [1,2]. Although infection is usually self-limiting in healthy individuals, children, the elderly and immunocompromised individuals have a higher risk of more serious and prolonged disease [3]. Novel norovirus genotypes and variants periodically emerge, replacing previously dominant strains, and most individuals experience multiple infections throughout their lifetime. Despite this significant health burden, no specific antiviral treatments or licensed vaccines are currently available, although several vaccine candidates are in clinical trials [1,4].

The norovirus genome comprises three open reading frames (ORFs). ORF2 and ORF3 encode the major (VP1) and minor (VP2) capsid proteins. VP1 is the most exposed and antigenically relevant structural protein of the virus, directly interacting with histo-blood group antigens (HBGAs), a family of glycans that are considered initial attachment factors of noroviruses. HBGAs determine the ABO and Lewis blood groups and are expressed on the surface of specific cell types (*e.g.*, enterocytes and red blood cells) and secreted in saliva and other bodily secretions [5]. The recognition of HBGAs by noroviruses is strain specific. Non-secretors, individuals lacking a functional fucosyltransferase 2 (FUT2) enzyme, express a restricted selection of HBGAs in the intestine and have shown resistance to infection by several strains [6,7]. In its native state, VP1 self-assembles into virus-like particles (VLPs) that mimic the HBGA binding specificity and antigenic properties of norovirus virions [8] and are commonly used to assess antibody-mediated blocking of HBGA binding. An alternative approach to norovirus VLPs for measuring antibody mediated blocking is the use of VP1 fused to a luciferase protein, whose binding to HBGAs is quantified by measuring luminescence, or the use of human intestinal enteroids [9,10].

Based on ORF2 sequence diversity, noroviruses are classified into ten genogroups, which are further subdivided into 48 genotypes [11]. Among circulating strains, the GII.4 Sydney variant, which emerged in 2012, was the predominant variant for over a decade [12]. GII.4 noroviruses accumulate amino acid substitutions in VP1, leading to the recurrent emergence of novel variants, whereas non-GII.4 noroviruses exhibit prolonged co-circulation with limited sequence variation [13,14]. In addition to the GII.4 Sydney variant, the GII.17 genotype has frequently been detected. Initially identified in 1978, GII.17 circulated for decades with sporadic notifications until the winter of 2014/2015, when clade D GII.17 emerged across multiple regions, including China, Taiwan, and Japan, with subsequent detections in Australia, France, Italy, the Netherlands, New Zealand, and Russia [15–19]. Environmental surveillance had detected GII.17 in river water from both rural and urban areas in Kenya as early as 2012/2013, and a few cases were reported in 2012 in South Korea [20,21]. Since its emergence, GII.17 has remained one of the most frequently detected genotypes [22].

Compared to earlier GII.17 strains, clade D GII.17 harbors substitutions in four putative antigenic epitopes, suggestive of immune evasion [23]. Additionally, this variant has a broad saliva-binding profile [23–25]. The GII.17 VP1 sequence has remained relatively conserved since the emergence of the clade D variant. However, during the winter of 2023/2024, a surge in GII.17 norovirus cases was observed across Austria, Germany, France, Ireland, the Netherlands, England, Brazil, and the United States, where it became the predominant genotype in several regions [26,27]. Phylogenetic analysis showed that sequences from these outbreaks were most similar to those of a large outbreak in Romania in 2021 [28]. This marked the first instance since the 2014/2015 winter season in which the GII.17 genotype has surpassed GII.4 Sydney in prevalence. A recent study showed that viruses from clades C, D, and the new clade bind a broader range of salivary HBGAs than those from clades A and B [25]. It also showed, using mouse sera, that the new clade was antigenically distinct from the other GII.17 variants, but most similar to clade C [25]. The underlying mechanisms driving the emergence of this novel GII.17 variant in 2024 may therefore involve an interplay between immune evasion, HBGA binding, and/or differences in the function of the nonstructural proteins.

Here, we investigated whether the recent emergence of GII.17 is associated with changes in HBGA binding and antigenicity in the context of human immunity. To this end, we selected both the novel GII.17 variant and other GII.17 strains for comparative analysis. We assessed HBGA binding using a broad panel of saliva samples (n = 12), synthetic glycans, and intestinal tissues from the duodenum, jejunum, and ileum. Additionally, binding-blocking assays with a large panel of human sera (n = 75) were performed to evaluate potential antigenic differences that may facilitate immune evasion.

## Results

### Genetic diversity of GII.17 VP1 and strain selection

**Fig 1:**
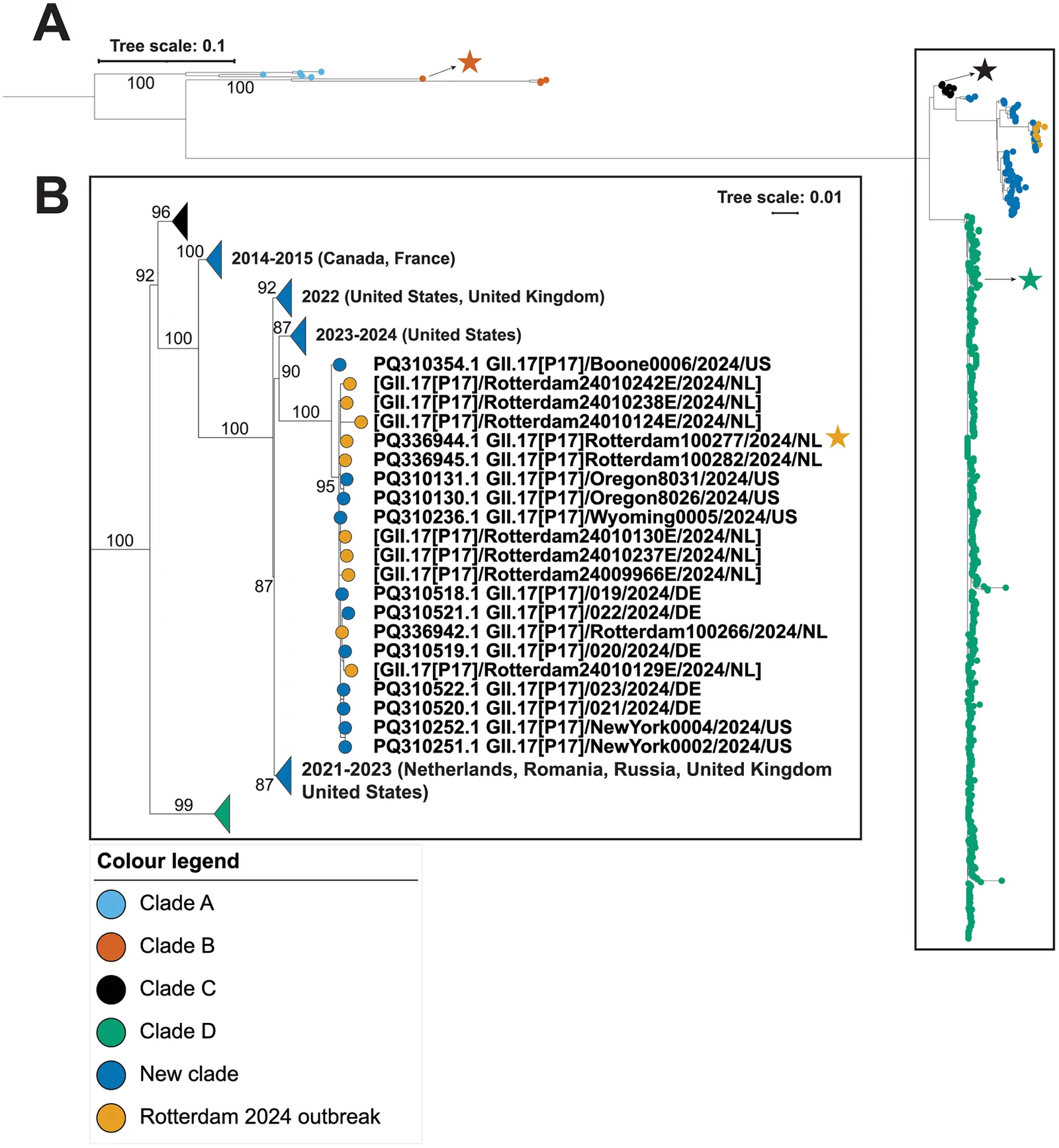
Phylogenetic analysis of GII.17 strains from 1978–2024. ORF2 (VP1) nucleotide sequences from GenBank (n = 621) were analyzed together with VP1 nucleotide sequences from norovirus-positive stool samples collected during an outbreak in Rotterdam, Netherlands (n = 10). A maximum-likelihood tree was constructed in IQ-TREE using the TIM2e+I + G4 model with 1,000 bootstrap replicates. **(A)** Overview of the complete phylogenetic tree, with clades A (light blue), B (dark orange), C (black), D (green), and the new clade (dark blue) indicated. Sequences from the outbreak in Rotterdam are highlighted in light orange. The inset rectangle indicates the region shown in detail in **(B)**. **(B)** Expanded view of the subclade within the new GII.17 clade containing the sequences from the outbreak in Rotterdam, with country and year of detection indicated. Stars denote strains selected for further testing: GII.17 2005 (dark orange star), GII.17 2014 (black star), GII.17 2015 (green star) and GII.17 2024 (orange star).

To visualize the GII.17 genetic diversity and select reference strains, we inferred a phylogenetic tree after aligning 621 GII.17 VP1 nucleotide sequences from GenBank and ten VP1 sequences from norovirus-positive stool samples from a large outbreak in Rotterdam, Netherlands, in 2024 (Fig 1A, S1 Data).

The sequences grouped in the previously described four clades (A-D) and the recently emerged new clade [25]. Most sequences belonged to the Kawasaki308 cluster (clade D), which was the dominant lineage for over 8 years. As previously reported by several groups, the sequences associated with the increase in detection of the GII.17 genotype in 2023–2024 (new clade) were genetically closer to the Kawasaki323 cluster (clade C), which co-circulated with clade D in 2014 but hadnot been reported since [25,28,29]. All 10 sequences from the 2024 Rotterdam outbreak clustered within a subclade of the new clade, which also included sequences from Germany and the USA from the same year (Fig 1B). To characterize the binding and antigenic properties of the GII.17 genotype per clade, we selected sequences representing four of the five GII.17 clades: a clade B strain (GII.17 2005), a clade C strain (GII.17 2014), a clade D strain (GII.17 2015), and a strain from the Netherlands that belongs to the new GII.17 clade (GII.17 2024).

### New clade GII.17 contains multiple amino acid substitutions in antigenic domains and HBGA binding sites

**Fig 2:**
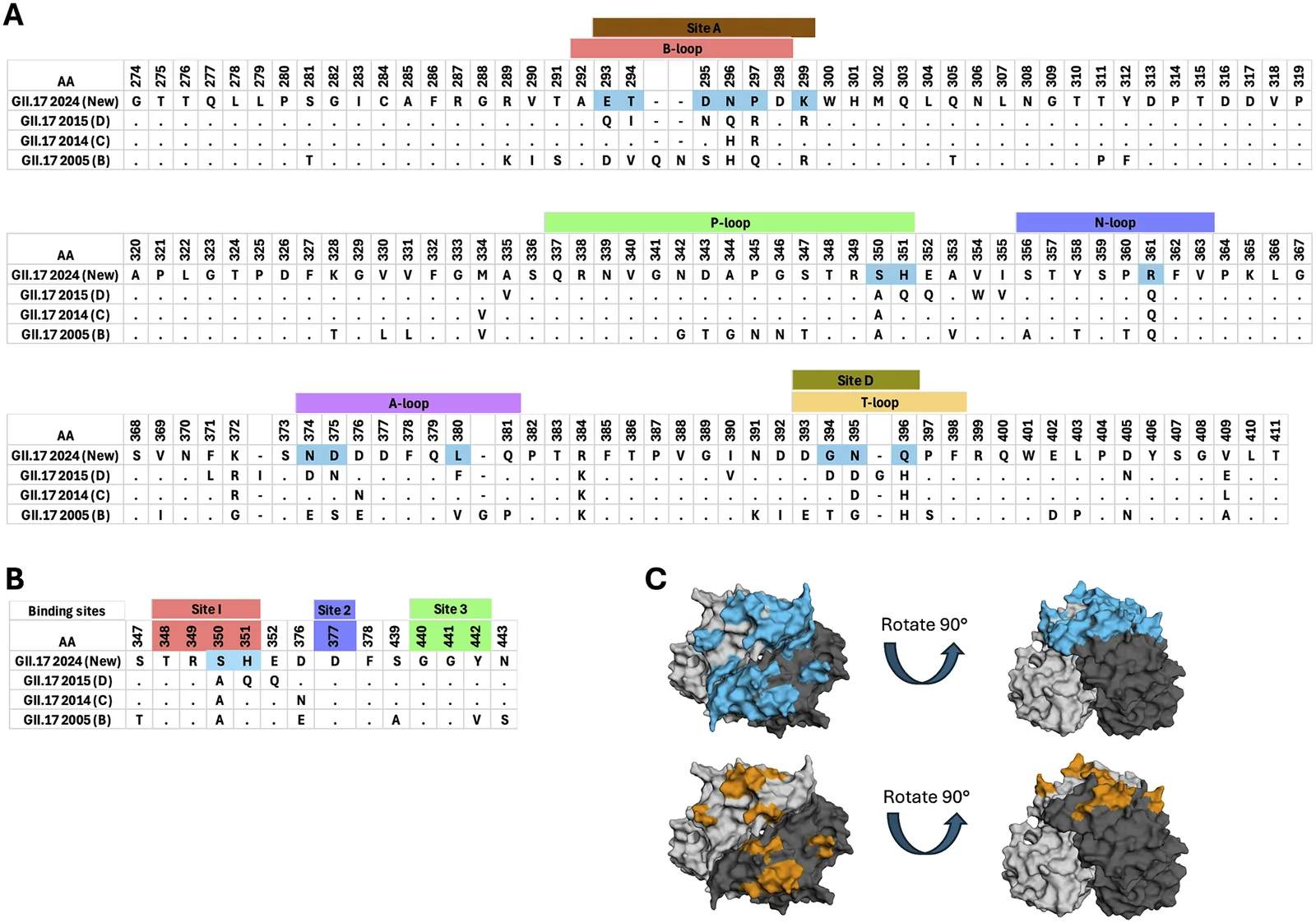
Mapping of amino acid differences in the P2 domain of GII.17 strains. Alignment of (A) the surface exposed loops and antigenic sites and (**B**) the HBGA binding sites of the selected GII.17 strains. Amino acid variations between GII.17 2024 and GII.17 2015 are marked in blue. **(C)** The GII.17 2024 P-dimer structure was modelled using SWISS-MODEL, with the GII.17 Kawasaki 323 P-dimer (PDB ID: 5f4m.1) as the template. Loops containing the HBGA binding interface and antigenic sites are indicated in blue, while differences between GII.17 2024 and GII.17 2015 are highlighted in orange.

We first investigated potential changes in the antigenic domains and HBGA binding sites of the new GII.17 2024 strain compared to other GII.17 variants (Fig 2). To address this, we performed a comparative analysis of the VP1 amino acid sequences from the four previously selected GII.17 norovirus strains. The experimentally defined residues corresponding to the partially characterized GII.17 antigenic sites A and D are indicated, together with the surrounding surface-exposed loops of the P2 subdomain. These loops contain or flank the major antigenic sites described for GII.4 noroviruses and are thought to represent key regions involved in antibody recognition (Fig 2A and 2C) [23,25,30–32]. Compared to GII.17 2015, the GII.17 2024 strain contained several amino acid substitutions and deletions. In antigenic site A, 6 of the 7 amino acids differed, while 4 out of 5 amino acids differed for site D. Fewer differences were observed in the A-loop, N-loop and the P-loop. Interestingly, the GII.17 2024 strain showed fewer amino acid differences in the P2 domain relative to GII.17 2014 than to GII.17 2015, whereas GII.17 2005 displayed the greatest divergence among the strains analyzed. Notably, although our GII.17 2024 reference strain encodes an N at position 395, several sequences within the new clade encode a D at this position.

For the HBGA binding sites, we referenced the locations identified by Qian *et al.* [33] (Fig 2B). When comparing new clade strain GII.17 2024 to clade D strain GII.17 2015, we observed two substitutions in binding site I at amino acids 350 and 351, and no changes in sites II and III. The alanine-to-serine substitution introduces a hydroxyl group potentially capable of forming new hydrogen bonds with neighboring residues or directly with HBGA sugars, potentially modulating binding affinity. Compared to GII.17 2014, GII.17 2024 had one amino acid difference in site I at position 350 and carried an asparagine-to-aspartate substitution adjacent to site II. This replacement of a neutral polar side chain with a negatively charged residue may alter existing hydrogen bond networks or local electrostatic interactions near the binding pocket. However, approximately half of the new GII.17 clade viruses still encode asparagine at this position. In contrast, GII.17 2005 differed more substantially: at site III, valine, a small hydrophobic residue, is present instead of tyrosine. Therefore, the binding pocket does not contain the aromatic ring and hydroxyl group of tyrosine, potentially causing reduced hydrogen bonding capacity. A tyrosine at this location was previously identified as a determinant of binding to A, B, and H antigens [25,33–35]. In addition, GII.17 2005 carried four further unique amino acids adjacent to binding sites, making it the most divergent strain with respect to the HBGA-binding interface.

We hypothesized that the observed changes in the antigenic and HBGA binding regions may influence HBGA-binding specificity and/or immune evasion, potentially enhancing viral spread.

### Attachment of GII.17 strains to HBGAs

**Fig 3:**
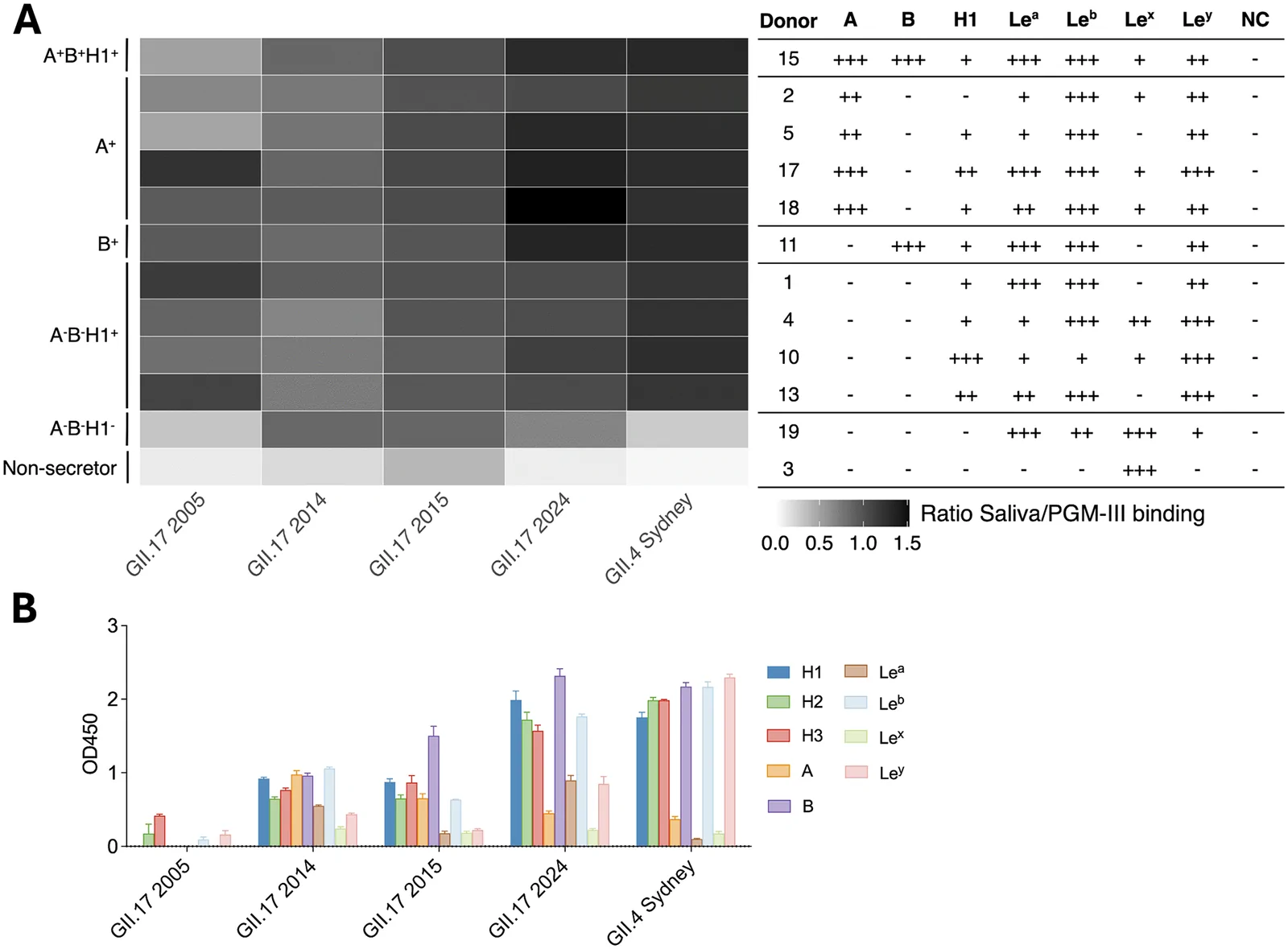
Binding of fluorescein isothiocyanate (FITC)-labelled virus-like particles (VLPs) to pig gastric mucin III (PGM-III), human saliva, and synthetic carbohydrates. **(A)** Binding of FITC-labelled VLPs to human saliva samples. VLP binding was detected using an anti-FITC antibody. Data represent mean absorbance values at 450 nm, normalized to PGM-III binding. Each assay was performed in duplicate. FITC-labelled GII.4 Sydney VLPs were included as a positive control for binding. Glycan abundance in saliva donors was defined as follows: (+) OD > 0.18 and < 0.5, (++) OD ≥ 0.5 and < 1.0, and (+++) OD ≥ 1.0. **(B)** Binding of FITC-labelled VLPs to synthetic carbohydrates**.** Plates were coated with PAA-conjugated HBGAs, and VLP binding was detected using an anti-FITC antibody. Data represent mean absorbance values at 450 nm after subtraction of background signals from the negative glycan control (D-mannose). Each assay was performed in duplicate. GII.4 Sydney VLPs were included as a positive control for binding. Le = Lewis.

To investigate the HBGA binding specificity of the GII.17 strains, we generated fluorescein isothiocyanate (FITC)-labelled VLPs of the four selected GII.17 strains and a GII.4 Sydney strain, which was included as a positive control due to its broad HBGA binding profile and high prevalence in the population. An empty expression plasmid was used to generate a negative control. We assessed the relative binding capacity of the FITC-VLPs to HBGAs in pig gastric mucin type III (PGM-III) and 12 saliva samples from healthy donors with diverse HBGA profiles (Fig 3A). The saliva samples represented individuals with AB, A, B, H1, and non-secretor (Se^-^) HBGAs. All strains displayed robust binding to PGM-III, supporting its use both for normalization in the binding assay and as the binding substrate in the binding-blocking assays.

All GII.17 strains bound to most saliva samples, while binding was not observed for non-secretor (Le^x^) saliva. GII.17 2005 showed reduced binding (*i.e.*, less than half of the PGM-III signal) to three additional saliva samples. No significant binding differences were observed among GII.17 2024, GII.17 2015 and GII.17 2014. The GII.4 Sydney VLPs included as a control bound to all saliva samples except the nonsecretor and one sample expressing only Le^a^, Le^b^, Le^x^ and Le^y^. No binding was detected for the FITC-labelled negative control.

Because saliva contains a complex mixture of HBGAs and other glycans, we next characterized VLP-HBGA interactions using individual synthetic HBGAs (Fig 3B). D-mannose was included as a negative control, as the VLPs were not expected to bind this carbohydrate. The VLP-HBGA interaction profiles varied among strains. GII.4 Sydney exhibited a strong and broad binding pattern, interacting with all tested HBGAs, although binding to Le^a^ and Le^x^ was low. GII.17 2024 displayed stronger overall binding, but its binding profile closely resembled those of GII.17 2015 and GII.17 2014, interacting strongly with all HBGAs except Le^x^. For GII.17 2015, binding to Le^a^ and Le^y^ was also low. GII.17 2005 had the narrowest binding profile, binding exclusively and weakly to H2, H3, Le^b^ and Le^y^.

In conclusion, although we observed differences in HBGA binding among GII.17 strains, these variations are unlikely to explain the increased prevalence of GII.17 2024. The binding profile of GII.17 2024 closely resembled that of GII.17 2014 and GII.17 2015 and did not indicate a substantial enhancement in HBGA interaction compared to earlier strains, except for GII.17 2005.

### FITC-labelled GII.17 VLPs bind to human intestinal tissues with variable affinities

To assess whether variations in HBGA binding specificity among GII.17 strains were reflected in their attachment to human intestinal tissues, we performed virus immunohistochemistry using FITC-labelled VLPs of the GII.17 and the GII.4 Sydney strains. The presence and integrity of VLPs was confirmed by electron microscopy (S1 Fig). Controls included VLPs produced from an empty expression vector (negative control) and unlabelled GII.4 Sydney VLPs (label control). Binding was evaluated on three duodenal, two jejunal, and three ileal tissues obtained from different donors. HBGA expression on the epithelial surfaces was characterized by immunohistochemistry.

**Fig 4:**
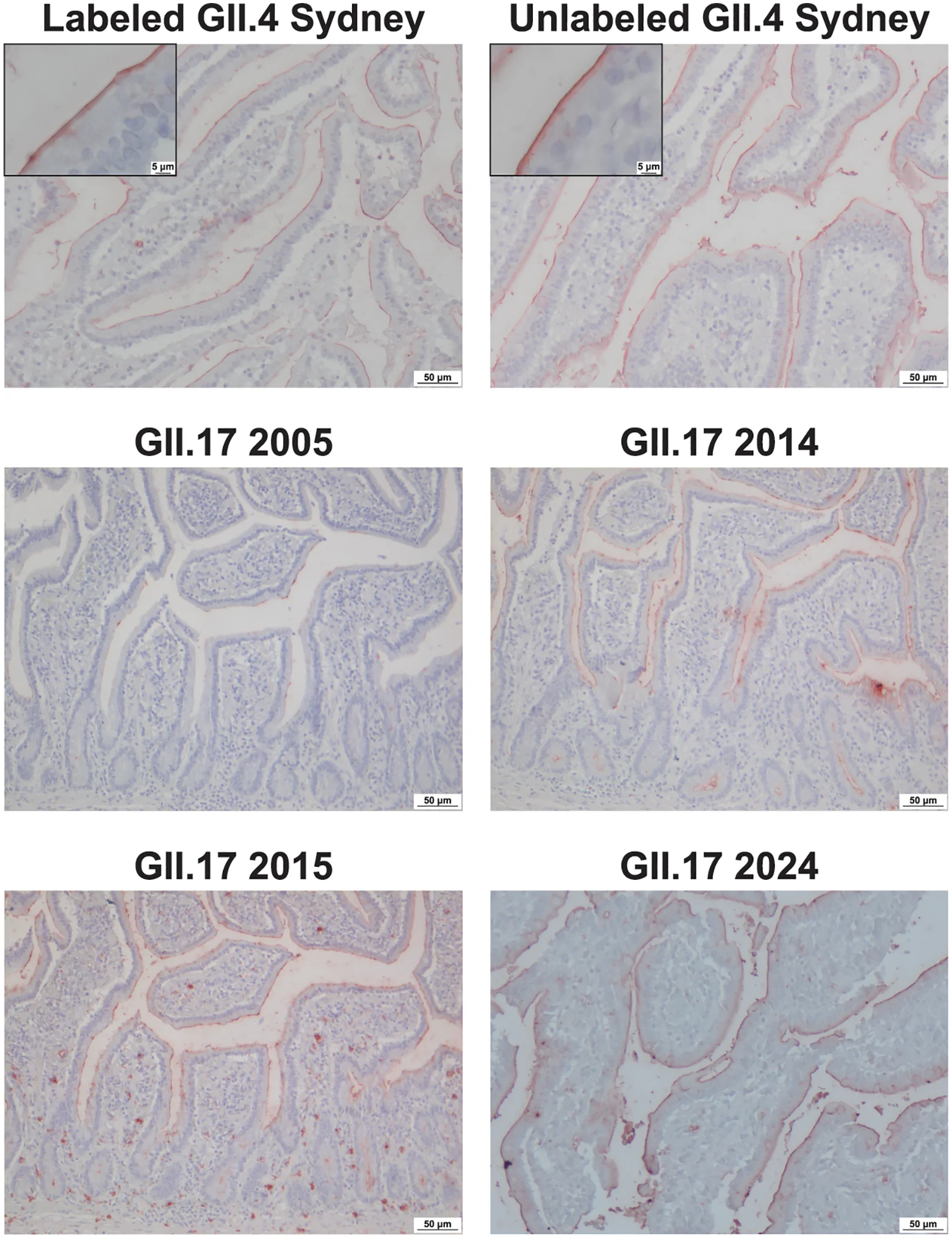
Attachment of GII.17 and GII.4 Sydney virus-like particles (VLPs) to human intestinal tissues. Binding of FITC-labelled and unlabelled GII.4 Sydney VLPs to human duodenal tissue (Le^a,b,x^), detected using anti-FITC and anti-norovirus antibodies, respectively. Comparative attachment of the FITC-labelled GII.17 VLPs to human duodenum expressing H2 and Lewis antigens (H2, Le^a,b,x^). Magnification, 10x and 100x (insets).

The included tissues exhibited distinct HBGA expression patterns on their epithelial surfaces, which are summarized in Table 1. Unlabelled and FITC-labelled GII.4 Sydney VLPs attached to the intestinal epithelium with similar efficiency (Fig 4 and S2), indicating that the FITC label did not interfere with tissue attachment. No epithelial staining was observed for the negative control.

**Table 1:** Immunohistochemistry results.

Small intestine section	Histo-blood group antigens (HBGAs)	Norovirus virus-like particles (VLPs)				
		GII.17 2005	GII.17 2014	GII.17 2015	GII.17 2024	GII.4 Sydney
Duodenum1	A, H2, Le^b^	–	+	+	+	+
Duodenum2	H2, Le^a,b,x^	+	+	+	+	–
Duodenum3	Le^a,b,x^	+	+	+	+	+
Jejunum1	A, B, Le^b^	+	+	+	+	+
Jejunum2	A, H2, Le^b^	–	+	+	+	+
Ileum1	A, B, Le^a,x,y^	–	–	–	–	–
Ileum2	B, Le^b^	+	+	+	+	+
Ileum3	H2, Le^a,b,y^	+	+	+	+	+

Ileum1 (A, B, Le^a,x,y^) was the only tissue to which none of the VLPs bound. Compared with the other GII.17 variants, GII.17 2005 bound fewer intestinal tissues and generally showed weaker binding, although it exhibited stronger attachment to Ileum2 (B, Le^b^). GII.17 2014, 2015, and 2024 showed similar staining intensities and interacted with all examined tissues except Ileum1. GII.4 Sydney displayed strong attachment to all tissues except Duodenum2 (H2, Le^a,b,x^) and Ileum1. Interestingly, no clear correlation between VLP attachment and HBGA expression profiles was observed.

In conclusion, the binding of the GII.17 VLPs to intestinal tissues was highly variable and we found no evidence for broadened receptor binding for GII.17 2024 compared to the GII.17 2014 and 2015 variants.

### Differences in antigenic properties between GII.17 strains

**Fig 5:**
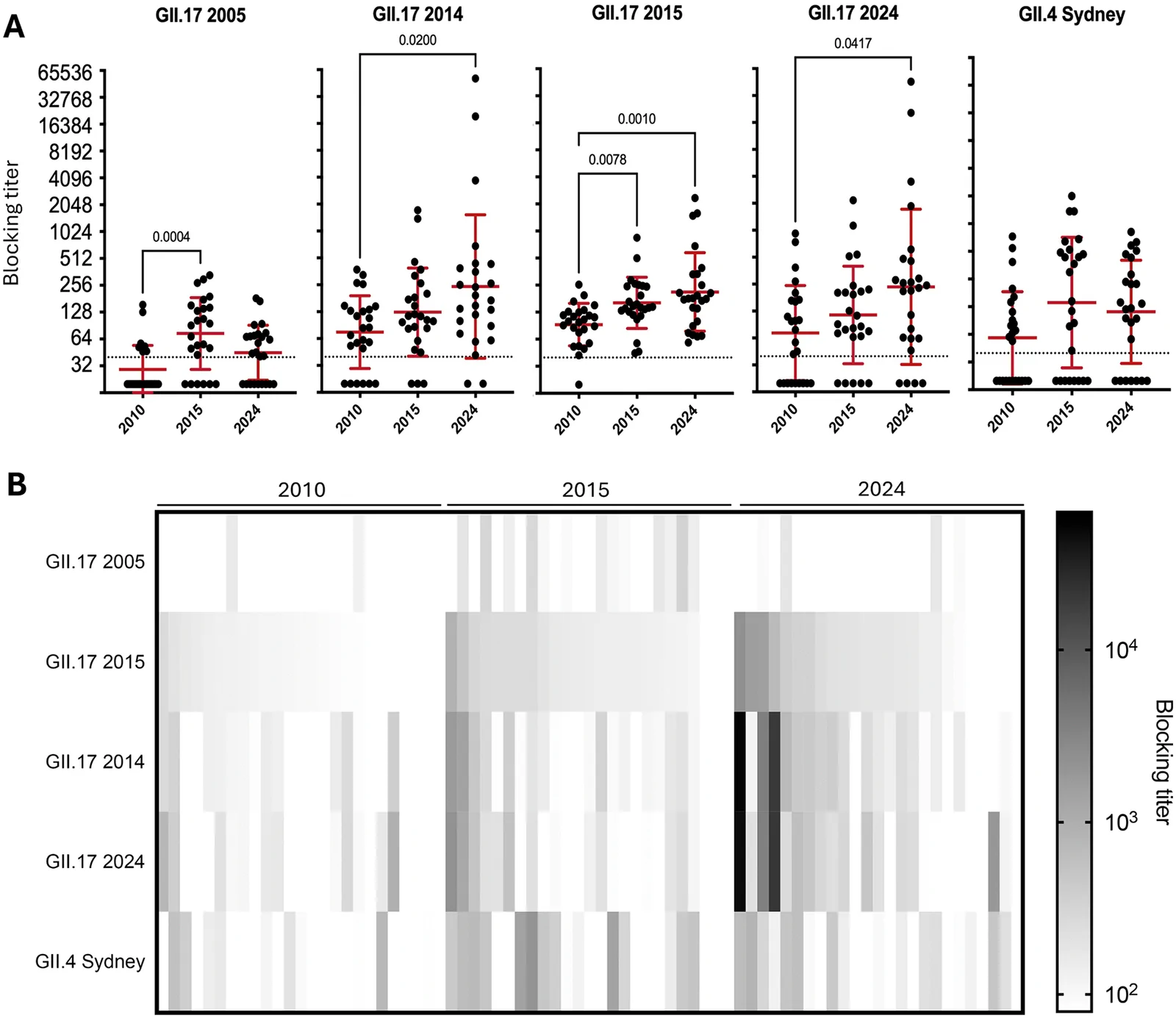
Serum blocking titers of the GII.17 and GII.4 Sydney NLuc-VP1 fusion proteins. A total of 75 serum samples, collected across different years, were assessed for their ability to inhibit the binding of NLuc-VP1 proteins to PGM-III. Serial dilutions were performed from 1:40 to 1:40,960, and samples with titers <40 were assigned a value of 20. **(A)** Each dot represents the blocking titer of an individual serum sample against a GII.17 or GII.4 Sydney NLuc-VP1 fusion protein. Data were grouped by serum collection year to highlight differences in blocking titers over time. Only statistically significant differences, as determined by the Kruskal–Wallis test followed by Dunn’s multiple comparisons test on log_2_-transformed values, are indicated. The dotted line indicates the cut-off value of 40. **(B)** A heatmap displaying the same serum blocking titers. Each column corresponds to an individual serum sample, and sera were sorted from highest to lowest mean blocking titer within each serum group. Color intensity reflects blocking titer levels; white indicates titers below 1:40.

We hypothesized that the recent increase in detection of GII.17 2024 may be partially driven by immune evasion, resulting from antigenic differences between this strain and previously circulating GII.17 variants. To investigate this, we selected serum sets representing different exposure histories to GII.17 variants. We performed a luciferase immunoprecipitation system (LIPS)-blocking assay (Fig 5A) using sera from healthy individuals collected during the winter seasons of 2010, 2015, and 2024 (n = 25 per year). The 2024 samples correspond to the period with large new clade outbreaks, while the 2015 samples reflect a time of widespread clade D circulation. The 2010 samples represent the pre-emergence phase of GII.17. We included the same GII.17 strains as those used in the binding studies, alongside GII.4 Sydney. GII.4 has been the predominant norovirus genotype over the past decade, and GII.4 Sydney has circulated since 2012. Blocking titers were defined as the serum dilution at which the relative light units (RLU) were reduced to 50% of the no-serum control, with higher titers indicating greater blocking activity.

Blocking titers differed significantly among sera collected in 2010, 2015, and 2024 for all GII.17 variants, while titers against GII.4 Sydney remained similar across the panels. For all GII.17 variants, the 2010 serum panel showed the lowest blocking titers, consistent with the limited circulation of GII.17 during this period. Conversely, for all GII.17 variants except GII.17 2005, the 2024 serum panel exhibited the highest titers. Only sera from 2015 displayed elevated blocking titers against GII.17 2005.

To further explore antigenic relationships among GII.17 strains, we visualized individual serum-antigen blocking titers using a heat map (Fig 5B). The binding-blocking profiles of the individual sera were most similar for GII.17 2024 and GII.17 2014. There were also sera that exhibited high titers against GII.17 2024 but not against GII.17 2014 and vice versa. Distinct patterns were also observed between GII.17 2024 and GII.17 2015, with some sera showing strong blocking activity against only one of these strains. This suggests that while GII.17 2024 likely shares antigenic features with GII.17 2014, it also possesses unique properties differentiating it from both GII.17 2014 and GII.17 2015. GII.17 2005 and GII.4 Sydney showed distinct binding-blocking titers for individual sera compared to the other strains.

In conclusion, our findings indicate that in the context of sera from healthy adult individuals who had likely experienced multiple norovirus infections [36], GII.17 2024 exhibits antigenic divergence from previously circulating GII.17 strains, with the closest relationship observed with GII.17 2014. This is consistent with our analysis of the putative antigenic domains. We propose that the recent surge in GII.17 2024 circulation is driven, at least in part, by immune evasion of this emerging variant.

## Discussion

The re-emergence of the GII.17[P17] genotype in 2023/2024, nearly a decade after its initial emergence in 2014/2015, underscores its epidemiological significance. To elucidate potential mechanisms underlying the recent surge in GII.17 detection, we compared binding properties of GII.17 2024 with those of previously circulating variants. By incorporating a panel of individual synthetic glycans rather than relying solely on complex biological substrates such as saliva, our study provides a higher-resolution assessment of HBGA specificity.

Reduced binding of the GII.17 2005 variant compared with the other GII.17 variants was consistently observed across experiments. All GII.17 variants bound strongly to a broad range of synthetic glycans, apart from GII.17 2005. Both GII.17 2014 and GII.17 2024 bound to Le^a^, confirming the findings of Tohma *et al.*, who used non-secretor saliva containing Le^a^ and low levels of Le^x^ [25]. Similarly, all GII.17 strains bound to saliva samples, particularly those containing A and B antigens, but not to saliva of a non-secretor containing Le^x^. Also here, GII.17 2005 bound to a more restricted panel of saliva. GII.17 2014, 2015 and 2024 showed broad attachment to tissues, although staining intensities varied. In contrast, GII.17 2005 exhibited generally weaker attachment than the other GII.17 variants, with strong binding observed for only one intestinal epithelium. The poor predictability of tissue attachment for GII.17 2005, based on the HBGA profile, may result from the structural diversity and complexity of glycans in saliva and tissues compared with synthetic glycans.

Previous work by us and others also demonstrated that GII.17 2005 attached to a more limited range of human intestinal tissues and HBGAs compared to GII.17 2014 and GII.17 2015 [24,33,37]. This reduced binding is explained by the presence of valine instead of tyrosine at aa 442 (new clade numbering), which was shown to dramatically reduce HBGA binding [33–35]. Although the extensive genetic divergence between pre-2014 and post-2014 GII.17 variants likely conferred an evolutionary advantage in HBGA recognition, we showed that the new clade GII.17 2024 did not exhibit further enhancement in the binding of the tested HBGA structures or intestinal tissues relative to clade C and clade D GII.17.

Beyond HBGA binding, we compared the antigenic properties of the GII.17 2024 and the other GII.17 variants. Our finding that new clade GII.17 is antigenically closest to clade C GII.17 2014 is supported by a recent study using mouse sera [25]. One strength of our analysis is the use of an extensive panel of human sera, enabling robust antigenic comparisons across GII.17 variants in the context of human immunity shaped by prior exposures. This expands upon previous work, where limited serum availability hindered the ability to detect antigenic differences [25]. Unlike GII.4 noroviruses, which exhibit a dynamic pattern of antigenic drift reminiscent of the epochal evolution observed in influenza A viruses, non-GII.4 genotypes typically demonstrate more constrained antigenic evolution, with some lineages persisting for decades with only minimal variation in the capsid protein VP1 [14,38]. In GII.4, the successive emergence of new variants is primarily driven by immune evasion via amino acid substitutions within antigenic sites A–G of the P2 domain. These regions are critical for antibody-mediated blockade of HBGA binding, thereby preventing infection [30,39–42]. Historically, GII.17 has displayed more restricted diversity, with five major phylogenetic clusters identified to date, and variable levels of circulation [13,43]. However, epidemic GII.17 strains from 2014-2015 were previously shown to be antigenically distinct from earlier variants [31,44]. Interestingly, GII.4 Sydney 2012 blocking titers were similar across all three serum groups, despite the earliest group predating the emergence of this variant. This likely reflects cross-reactive immunity elicited by earlier GII.4 variants and/or immune imprinting [45,46].

Several of the amino acid differences between GII.17 2024 and earlier GII.17 strains are located within site A, which has historically been the most immunodominant region for GII.4 [12]. Site D also contained amino acid substitutions and, like site A, has been shown to play an important role in GII.17 antigenicity [12]. It is also important to consider that GII.4 Sydney 2012 has been circulating for over a decade. As a result, herd immunity against GII.4 may be different now than it was during the initial emergence of GII.17 in 2014/2015. Therefore, the current immunological landscape may be more permissive for the spread of a novel genotype such as GII.17 2024.

Our study focused exclusively on the properties of the GII.17 capsid, but the emergence of GII.17[P17] may also have involved changes in ORF1. Historically, the GII.17 capsid was predominantly associated with GII.P16, GII.P31 and GII.P13. In contrast, the epidemic GII.17 lineage that emerged in 2014 was associated with the GII.P17 polymerase [15,16,47]. The GII.P17 RdRp may have conferred enhanced viral fitness, potentially through increased polymerase activity. Indeed, the RdRp activity of the epidemic GII.17[P17] strain was 2.5-fold higher than that of the non-epidemic GII.8[P8] [48]. The recently emerged GII.17[P17] strains also show ORF1 variation, giving rise to a distinct sub-lineage within the GII.17[P17] cluster [26], though whether this confers unique biological properties that contributed to its spread remains unclear. In addition to RdRp, other nonstructural proteins may have played a role [25].

In summary, we demonstrate that the re-emergence of GII.17[P17] in 2023–2024 is likely driven by antigenic drift, enabling immune escape. Although increased HBGA binding and tissue attachment may also contribute, they are unlikely to be the sole drivers. Whether GII.17 will continue to evolve with sustained epidemic potential, like GII.4, remains an open question for future surveillance and research.

## Materials & methods

### Ethics statement

Sera were obtained from the biobank of the department of Viroscience at Erasmus MC. Ethical approval for the use of the sera was obtained in MEC-2022–0675. Formal written consent was obtained for all serum samples. Human intestinal tissue samples were obtained from the Pathology Research and Trial Support (PARTS) biobank at Erasmus MC. Use of coded residual tissue was approved by the Erasmus MC Medical Ethics Committee/Non-WMO Review Committee (METC-2018–1307) and complied with Erasmus MC Central Biobank regulations.

### Phylogenetic analyses

Complete GII.17 VP1 nucleotide sequences were retrieved from GenBank and aligned using MAFFTv7 [49]. Maximum-likelihood trees were generated using IQ-TREE [50], with the TIM2e+I + G4 model and 1000 bootstrap replicates.

### Mapping of amino acid substitutions onto P dimer

Amino acid substitutions were mapped onto the 3D P-dimer structure using EzMol v2.1 [51]. The structure of the GII.17 strain was predicted by homologous modelling using SWISS-MODEL (https://swissmodel.expasy). The model was built based on a GII.17 P-dimer (PDB 5f4m.1) crystal structure. HBGA binding site locations were taken from Tan *et al.* [52].

### Plasmid constructs

VP1 sequences of GII.17 2005 (DQ438972), GII.17 2014 (AB983218), GII.17 2015 (KX424646) and GII.4 Sydney (MT232050) were previously cloned in pCAGGS [37]. The VP1 sequence of GII.17 2024 (PQ336944) was amplified from a stool sample and cloned into pCAGGS. To generate NLuc–VP1 fusion constructs, VP1 coding regions were amplified using primers containing XhoI and XbaI restriction sites and ligated into pNLuc. All plasmids were sequenced using Sanger or whole-plasmid sequencing (Plasmidsaurus).

### Cell culture

Human embryonic kidney cells (HEK293T) were cultured in Dulbecco’s modified Eagle’s medium (DMEM; Lonza) supplemented with 10% fetal bovine serum (FBS, Sigma-Aldrich), nonessential amino acids (Lonza), Pen/Strep (Lonza), L-Glutamine (Lonza), sodium pyruvate (Gibco), and gentamicin. Cells were cultured at 37°C with 5% CO_2_.

### NLuc-VP1 fusion proteins

Twenty-four hours prior to transfection, 3 × 10^6^ HEK293T cells were seeded in 10 cm plates in DMEM without Pen/Strep and supplemented with 10% FBS, non-essential amino acids, and sodium pyruvate. Cells were transfected using polyethyleneimine (PEI; Polysciences). The transfection mixture contained 10 µg of pNLuc-VP1 plasmid and 30 µl of PEI (1 µg/µl) in 1 ml OptiMEM (Gibco). Cells were lysed 48h post-transfection to harvest NLuc-VP1 fusion proteins, as described previously [53]. Expression of VP1 protein was confirmed by western blot using a primary mouse monoclonal anti-NLuc antibody (1:500, Promega), and a horseradish peroxidase (HRP) conjugated secondary rabbit anti-mouse antibody (1:10,000, Dako).

### HBGA-binding of VLPs to saliva-coated plates

Saliva samples were obtained from 12 healthy donors. Samples were centrifuged at 10,000 x g for 5 min, and the supernatant was boiled at 100°C for 5 min. Flat-bottom transparent 96-well EIA plates (Costar) were coated with PGM-III (100 µg/mL in PBS, Sigma), 1% BSA or saliva (1:500 in PBS) overnight at 4°C, followed by 3 washes with PBS-T. Plates were then blocked with blocking buffer at RT for 1h and washed. 50 ng FITC-labelled VLPs in 0.1% BSA in PBS-T were added in duplicate to the wells and incubated overnight at 4°C. FITC-labelled VLPs were detected with an HRP-conjugated anti-FITC antibody (1:1000; Dako), for 1h at 4°C. Signal was developed using the TMB 2‑component microwell peroxidase substrate kit (SeraCare), and the reaction was stopped after 10 min with 3 M H_2_SO_4_. Absorbance was measured at OD_450_, and the background signal was corrected by subtraction of the values obtained for the BSA control.

### LIPS-blocking assay using serum samples

To assess potential differences in the antigenic profiles, we analyzed sera from 75 individuals (>18 years). Serum samples from healthy donors were collected in January 2010, December 2015, and December 2024, with 25 samples per time point. Information regarding prior norovirus exposure and secretor status was not available. Antibody-blocking titers were measured using the LIPS-blocking assay with minor modifications [54]. Flat-bottom white 96-well Maxisorp plates (ThermoFisher) coated with PGM-III were blocked with blocking buffer. NLuc-VP1 lysates were diluted to 1x10^5^ RLU/µl in blocking buffer to standardize RLU input. Sera were diluted 4-fold in blocking buffer, starting with a 1:40 dilution, and preincubated with NLuc-VP1 (1:1 volume) in a round-bottom 96-well plate for 2 h at 37°C while rocking. 100 µl antibody-NLuc-VP1 mixtures were added in duplicate to PGM-III-coated wells and incubated for 1 h at 37°C while rocking. Plates were washed and the bound antigen was detected as described before. The blocking titer was defined as the serum dilution at which the RLU value was 50% of the no serum control. A value of 20 was assigned to samples that did not show at least a 50% reduction in luciferase activity compared to the no serum control. IC_50_ values were determined using the nonlinear fit – one site log(IC_50_) binding curve analysis (top constraint = 100, bottom constraint = 0). For the blocking assays, data distribution was evaluated using the Shapiro-Wilk test. As not all datasets followed a normal distribution, statistical differences between groups with titers against the same NLuc–VP1 fusion proteins were assessed using the Kruskal–Wallis test followed by Dunn’s multiple comparisons test on log_2_-transformed values. A p-value < 0.05 was considered statistically significant. All statistical analyses were conducted using GraphPad Prism v. 10.4.

### Enzyme-linked immunosorbent assay (ELISA)-based carbohydrate assay

Streptavidin coated high capacity plates (Pierce, Invitrogen) were coated overnight at 4°C with biotinylated synthetic oligosaccharides (10 μg/ml; Table 2) conjugated to polyacrylamide (PAA) and eluted in 1 × TBS buffer (20 mM Tris, 150 mM NaCl, pH 7.2). Plates were washed five times with cold PBS and subsequently blocked at RT with 5% BSA in PBS. After blocking, and between all incubation steps, plates were washed five times with 0.01% Tween 20 in PBS (PBS T). One hundred nanograms of FITC-labelled VLPs in 0.1% BSA in PBS-T was added and incubated overnight at 4°C. FITC-labelled VLPs were detected with an HRP-conjugated anti-FITC antibody (1:1000; Dako), for 1h at 4°C. Signal was developed using the TMB 2 component microwell peroxidase substrate kit (SeraCare), and the reaction was stopped after 10 min with 3 M H_2_SO_4_. Absorbance was measured at OD_450_, and the background signal was corrected by subtraction of the values obtained for the negative control (D-mannose). Each VLP was tested in duplicate.

**Table 2:** Synthetic HBGA structures used for the glycan binding assay.

Name	Structure	Category	Manufacturer
Lewis *d* (H type 1)-PAA-biotin	Fucα1-2Galβ1-3GlcNAcβ1	Trisaccharide	Carbosynth
H (type 2)-PAA-biotin	Fuca1-2Galβ1-4GlcNAcβ1	Trisaccharide	Glycotech
H (type 3)-PAA-biotin	Fucα1-2Galβ1-3GalNAcα1	Disaccharide	Glycotech
Blood type A (tri)-PAA biotin	GalNAcα1-3(Fucα1-2)Galβ1	Trisaccharide	Glycotech
Blood type B (tri)-PAA biotin	Galα1-3(Fucα1-2)Galβ1	Trisaccharide	Glycotech
Lewis *a*-PAA-biotin	Galβ1-3(Fucα1-4)GlcNAcβ	Trisaccharide	Glycotech
Lewis *b*-PAA-biotin	Fucα1-2Galβ1-3(Fucα1-4)GlcNAcβ	Tetrasaccharide	Glycotech
Lewis *x*-PAA-biotin	Galβ1-4(Fucα1-3)GlcNAcβ1	Trisaccharide	Glycotech
Lewis *y*-PAA-biotin	Fucα1-2Galβ1-4(Fucα1-3)GlcNAcβ1	Tetrasaccharide	Glycotech
α-D-Mannose-PAA-biotin	α-D-Man	Monosaccharide	GlycoNZ

Glc = glucose; Fuc = fucose; Gal = galactose; GlcNAc = N-acetylglucosamine; Lac = lactose; GalNAc = N-acetylgalactosamine; Man = Mannose.

### VLP production and FITC labelling

VLPs were produced in 293T cells as described previously [8,55]. Twenty-four hours before transfection, 3 x 10^6^ 293T cells were plated in gelatinized 10 cm-plates. The VP1-pCAGGS constructs and an empty pCAGGS were transfected with calcium phosphate. Sixty-four hours post-transfection, cells were harvested and centrifuged (235 x g, 10 min). The supernatant was kept on ice. The pellet was resolved in 4 ml lysis buffer (1% Triton X-100) with protease inhibitor (cOmplete Mini EDTA-free protease inhibitor cocktail; Roche) in PBS and kept on ice for 15 min. The supernatant was added to the lysate, and centrifuged (2113 x g, 15 min), after which the supernatant was centrifuged through a 20% (w/w) sucrose cushion (122,253 x g, 2 hours). The pellet was dissolved in 2 ml PBS for 20 min on ice and subsequently centrifuged through a 20% to 60% sucrose gradient (overnight, 171,725 x g at 4°C). One-ml fractions were collected and VP1 presence confirmed by SDS-PAGE. VP1-containing fractions were concentrated using a 100-kDa Amicon filter (Millipore). VLPs were labelled as previously described [56]. For quantification, FITC-labelled VLPs were run on a 10% SDS-PAGE gel with a BSA concentration marker. Silver staining (Pierce) was done according to the manufacturer’s instructions. VLP presence and integrity was confirmed by electron microscopy by the EM Core Facility of Leiden University Medical Centre.

### HBGA typing by immunohistochemistry (IHC)

Human intestinal tissues were kindly provided by the Pathology Research and Trial Service (PARTS) at Erasmus MC. Three µm thick formalin-fixed paraffin-embedded (FFPE) tissue slides were deparaffinized with xylene and hydrated using a graded ethanol series (100 > 100 > 95 > 90 > 70%). HBGA expression was assessed by IHC. To block endogenous peroxidase, slides were incubated with 3% H_2_O_2_ diluted in PBS at RT for 10 min. All antibody incubation steps were carried out in 0.1% BSA for 1h at RT with 2 washing steps with PBS-T in between. HBGAs were detected with primary antibodies against antigen A (1:1; 9113D10; Diagast), B (1:1; 9621A8; Diagast), AB (1:1; 9113D10 + 152D12; Diagast), Neg (1:1; Diagast), Le^y^ (1:50; H18A; Absolute Antibody), Le^a^ (1:50; 7-LE; Sigma-Aldrich), Le^b^ (1:50; 2–25LE; Sigma-Aldrich), Le^x^ (1:100; MC480; Thermo Fisher), H1 (1:100; 17–206; Thermo Fisher), and H2 (1:40; BRIC231; Santa Cruz Biotechnology). A secondary biotinylated rabbit anti-mouse (1:100; Dako) was used, followed by streptavidin-horseradish peroxidase (HRP)-conjugated antibody (1:300; Dako). Peroxidase was revealed with 3-amino-9-ethyl-carbazole (Sigma-Aldrich). Tissues were counterstained with hematoxylin and embedded in Meyer’s glycerol-gelatine (Merck).

### Virus histochemistry on tissue sections

Tissue slides were prepared as for IHC (described above). TNB (0.1 M Tris, 0.15 M NaCl, pH 7.5, with 0.5% blocking reagent; Perkin Elmer) was used as blocking buffer and to dilute all the antibodies. Slides were blocked for 30 min at RT, and 50 ng of VLPs or 10 µl negative control were added and incubated overnight at 4°C. Between all subsequent steps, slides were washed twice with PBS-T, and all incubation steps were done at RT. FITC-labelled VLPs were detected by peroxidase-labelled rabbit anti-FITC (1:100; Dako) for 1 h. The signal was amplified using a tyramide signal amplification system (Perkin Elmer) according to the manufacturer’s instructions. Streptavidin-HRP was added at 1:300 and incubated for 30 min. As a control, unlabelled GII.4 Sydney VLPs were used and stained with a primary anti-GI/GII antibody (1:100; MAB242P; BBI Solutions).

## Supporting information

S1 Fig Electron microscopy images of norovirus virus-like particles (VLPs) used in this study. Magnification: GII.17 VLPs 40,836x, GII.4 Sydney VLPs 20,418x. Pixel size: GII.17 VLPs 0.37 nm, GII.4 Sydney VLPs 0.74 nm.
(TIF)

S2 Fig Attachment of GII.17 and GII.4 Sydney virus-like particles (VLPs) to human intestinal tissues. Binding of FITC-labelled and unlabelled GII.4 Sydney VLPs to human jejunal (A) and ileal (B) tissues, detected using anti-FITC and anti-norovirus antibodies, respectively. Comparative attachment of the FITC-labelled GII.17 VLPs to human jejunum (A, B, Le^b^) and ileum (B, Le^b^). Magnification, 10x and 100x (insets).
(TIF)

S1 Data Alignment of complete GII.17 ORF2 (VP1) nucleotide sequences. Sequences were retrieved from GenBank (n = 621) and norovirus-positive stool samples collected during an outbreak in Rotterdam, Netherlands (n = 10) were included.
(FASTA)

## References

[1] de Graaf M, van Beek J, Koopmans MPG. Human norovirus transmission and evolution in a changing world. Nat Rev Microbiol. 2016;14(7):421–33. doi: 10.1038/nrmicro.2016.48 27211790

[2] Pires SM, Fischer-Walker CL, Lanata CF, Devleesschauwer B, Hall AJ, Kirk MD, et al. Aetiology-Specific Estimates of the Global and Regional Incidence and Mortality of Diarrhoeal Diseases Commonly Transmitted through Food. PLoS One. 2015;10(12):e0142927. doi: 10.1371/journal.pone.0142927 26632843 PMC4668836

[3] Trivedi TK, Desai R, Hall AJ, Patel M, Parashar UD, Lopman BA. Clinical characteristics of norovirus-associated deaths: a systematic literature review. Am J Infect Control. 2013;41(7):654–7. doi: 10.1016/j.ajic.2012.08.002 23266383

[4] Prasad BVV, Atmar RL, Ramani S, Palzkill T, Song Y, Crawford SE, et al. Norovirus replication, host interactions and vaccine advances. Nat Rev Microbiol. 2025;23(6):385–401. doi: 10.1038/s41579-024-01144-9 39824927 PMC12088900

[5] Tenge VR, Hu L, Prasad BVV, Larson G, Atmar RL, Estes MK, et al. Glycan Recognition in Human Norovirus Infections. Viruses. 2021;13(10):2066. doi: 10.3390/v13102066 34696500 PMC8537403

[6] Nordgren J, Svensson L. Genetic Susceptibility to Human Norovirus Infection: An Update. Viruses. 2019;11(3):226. doi: 10.3390/v11030226 30845670 PMC6466115

[7] Hong X, Xue L, Liao Y, Wu A, Jiang Y, Kou X. Association of fucosyltransferase 2 gene with norovirus infection: A systematic review and meta-analysis. Infect Genet Evol. 2021;96:105091. doi: 10.1016/j.meegid.2021.105091 34610432

[8] Taube S, Kurth A, Schreier E. Generation of recombinant norovirus-like particles (VLP) in the human endothelial kidney cell line 293T. Arch Virol. 2005;150(7):1425–31. doi: 10.1007/s00705-005-0517-x 15789263

[9] Ettayebi K, Crawford SE, Murakami K, Broughman JR, Karandikar U, Tenge VR, et al. Replication of human noroviruses in stem cell-derived human enteroids. Science. 2016;353(6306):1387–93. doi: 10.1126/science.aaf5211 27562956 PMC5305121

[10] Izquierdo-Lara RW, Villabruna N, Hesselink DA, Schapendonk CME, Ribó Pons S, Nieuwenhuijse D, et al. Patterns of the within-host evolution of human norovirus in immunocompromised individuals and implications for treatment. EBioMedicine. 2024;109:105391. doi: 10.1016/j.ebiom.2024.105391 39396425 PMC11663770

[11] Chhabra P, de Graaf M, Parra GI, Chan MC-W, Green K, Martella V, et al. Updated classification of norovirus genogroups and genotypes. J Gen Virol. 2019;100(10):1393–406. doi: 10.1099/jgv.0.001318 31483239 PMC7011714

[12] Parra GI, Tohma K, Ford-Siltz LA, Eguino P, Kendra JA, Pilewski KA, et al. Minimal Antigenic Evolution after a Decade of Norovirus GII.4 Sydney_2012 Circulation in Humans. J Virol. 2023;97(2):e0171622. doi: 10.1128/jvi.01716-22 36688654 PMC9973034

[13] Parra GI, Squires RB, Karangwa CK, Johnson JA, Lepore CJ, Sosnovtsev SV, et al. Static and Evolving Norovirus Genotypes: Implications for Epidemiology and Immunity. PLoS Pathog. 2017;13(1):e1006136. doi: 10.1371/journal.ppat.1006136 28103318 PMC5283768

[14] Parra GI. Emergence of norovirus strains: A tale of two genes. Virus Evol. 2019;5(2):vez048. doi: 10.1093/ve/vez048 32161666 PMC6875644

[15] de Graaf M, van Beek J, Vennema H, Podkolzin AT, Hewitt J, Bucardo F, et al. Emergence of a novel GII.17 norovirus-End of the GII.4 era? Euro Surveill. 2015. doi: www.eurosurveillance.org:pii=21178 10.2807/1560-7917.es2015.20.26.21178 PMC5921880 26159308

[16] Matsushima Y, Ishikawa M, Shimizu T, Komane A, Kasuo S, Shinohara M, et al. Genetic analyses of GII.17 norovirus strains in diarrheal disease outbreaks from December 2014 to March 2015 in Japan reveal a novel polymerase sequence and amino acid substitutions in the capsid region. Euro Surveill. 2015;20(26):21173. doi: 10.2807/1560-7917.es2015.20.26.21173 26159307

[17] Lu J, Sun L, Fang L, Yang F, Mo Y, Lao J, et al. Gastroenteritis outbreaks caused by norovirus GII.17, Guangdong Province, China, 2014–2015. Emerg Infect Dis. 2015;21:1240–2. doi: 10.3201/eid2107.150226 26080037 PMC4480401

[18] Fu J, Ai J, Jin M, Jiang C, Zhang J, Shi C, et al. Emergence of a new GII.17 norovirus variant in patients in Jangsu, China, September 2014 to March 2015. Euro Surveill. 2015;20. 10.2807/1560-7917.es2015.20.24.21157 26111236

[19] Rackoff LA, Bok K, Green KY, Kapikian AZ. Epidemiology and evolution of rotaviruses and noroviruses from an archival WHO Global Study in Children (1976-79) with implications for vaccine design. PLoS One. 2013;8(3):e59394. doi: 10.1371/journal.pone.0059394 23536875 PMC3607611

[20] Kiulia NM, Mans J, Mwenda JM, Taylor MB. Norovirus GII.17 predominates in selected surface water sources in Kenya. Food Environ Virol. 2014;6:221–31. doi: 10.1007/s12560-014-9160-6 25059212

[21] Cho HG, Lee SG, Kim WH, Lee JS, Park PH, Cheon DS, et al. Acute gastroenteritis outbreaks associated with ground-waterborne norovirus in South Korea during 2008-2012. Epidemiol Infect. 2014;142(12):2604–9. doi: 10.1017/S0950268814000247 24534556 PMC9151260

[22] van Beek J, de Graaf M, Al-Hello H, Allen DJ, Ambert-Balay K, Botteldoorn N. Molecular surveillance of norovirus, 2005–16: an epidemiological analysis of data collected from the NoroNet network. Lancet Infect Dis. 2018;18:545–53. doi: 10.1016/S1473-3099(18)30059-8 29396001

[23] Chan MCW, Lee N, Hung T-N, Kwok K, Cheung K, Tin EKY, et al. Rapid emergence and predominance of a broadly recognizing and fast-evolving norovirus GII.17 variant in late 2014. Nat Commun. 2015;6:10061. doi: 10.1038/ncomms10061 26625712 PMC4686777

[24] Estienney M, Tarris G, Abou-Hamad N, Rouleau A, Boireau W, Chassagnon R, et al. Epidemiological Impact of GII.17 Human Noroviruses Associated With Attachment to Enterocytes. Front Microbiol. 2022;13:858245. doi: 10.3389/fmicb.2022.858245 35572680 PMC9094630

[25] Tohma K, Jacobsen S, Altmann B, Kendra JA, Landivar M, De La O WE. GII.17 norovirus re-emerged in the 2020s as a result of dynamic and adaptive evolutionary processes. Nat Commun. 2025. doi: 10.1038/s41467-025-66279-6 PMC12749941 41285779

[26] Chhabra P, Wong S, Niendorf S, Lederer I, Vennema H, Faber M. Increased circulation of GII.17 noroviruses, six European countries and the United States, 2023 to 2024. Eurosurveillance. 2024;29. doi: 10.2807/1560-7917.ES.2024.29.39.2400625 PMC11484341 39328162

[27] Malta FC, de Souza Mello M, Arantes I, de Andrade J da SR, Fialho AM, Baduy GA, et al. Emergence and predominance of GII.17[P17] noroviruses in Brazil, 2023–2024. Sci Rep. 2026. doi: 10.1038/s41598-026-49730-6 PMC13365198 42034845

[28] Dinu S, Oprea M, Iordache R-I, Rusu L-C, Usein C-R. Genome characterisation of norovirus GII.P17-GII.17 detected during a large gastroenteritis outbreak in Romania in 2021. Arch Virol. 2023;168(4):116. doi: 10.1007/s00705-023-05741-6 36947248

[29] Epifanova NV, Oparina SV, Morozova OV, Sashina TA, Alekseeva AE, Novikova NA. Appearance and spread of norovirus genotype GII.17 subcluster C2 (Romania-2021 like) in Nizhny Novgorod, Russia, 2021–2023. Arch Virol. 2025;170. doi: 10.1007/s00705-025-06356-9 40576844

[30] Tohma K, Lepore CJ, Gao Y, Ford-Siltz LA, Parra GI. Population Genomics of GII.4 Noroviruses Reveal Complex Diversification and New Antigenic Sites Involved in the Emergence of Pandemic Strains. mBio. 2019;10(5):e02202-19. doi: 10.1128/mBio.02202-19 31551337 PMC6759766

[31] Lindesmith LC, Kocher JF, Donaldson EF, Debbink K, Mallory ML, Swann EW, et al. Emergence of Novel Human Norovirus GII.17 Strains Correlates With Changes in Blockade Antibody Epitopes. J Infect Dis. 2017;216(10):1227–34. doi: 10.1093/infdis/jix385 28973354 PMC5853573

[32] Yi Y, Wang X, Wang S, Xiong P, Liu Q, Zhang C, et al. Identification of a blockade epitope of human norovirus GII.17. Emerg Microbes Infect. 2021;10(1):954–63. doi: 10.1080/22221751.2021.1925162 33929932 PMC8143627

[33] Qian Y, Song M, Jiang X, Xia M, Meller J, Tan M. Structural adaptations of norovirus GII.17/13/21 lineage through two distinct evolutionary paths. J Virol. 2019;93:1655–73. doi: 10.1128/JVI PMC6288326 30333166

[34] Singh BK, Koromyslova A, Hefele L, Gürth C, Hansman GS. Structural evolution of the emerging 2014-2015 GII.17 noroviruses. J Virol. 2016;90:2710–5. doi: 10.1128/jvi.03119-15 PMC4810701 26699640

[35] Tan M, Xia M, Cao S, Huang P, Farkas T, Meller J, et al. Elucidation of strain-specific interaction of a GII-4 norovirus with HBGA receptors by site-directed mutagenesis study. Virology. 2008;379(2):324–34. doi: 10.1016/j.virol.2008.06.041 18692213 PMC3503251

[36] Reyes Y, González F, Gutiérrez L, Blandón P, Centeno E, Zepeda O, et al. Secretor Status Strongly Influences the Incidence of Symptomatic Norovirus Infection in a Genotype-Dependent Manner in a Nicaraguan Birth Cohort. J Infect Dis. 2022;225(1):105–15. doi: 10.1093/infdis/jiab316 34129046 PMC8730499

[37] Villabruna N, Schapendonk CME, Aron GI, Koopmans MPG, De Graaf M, Pfeiffer JK. Human Noroviruses Attach to Intestinal Tissues of a Broad Range of Animal Species. 2021. doi: 10.1128/JVI.01492 PMC7925106 33115870

[38] Koelle K, Cobey S, Grenfell B, Pascual M. Epochal Evolution Shapes the Phylodynamics of Interpandemic Influenza A (H3N2) in Humans. Science. 2006;314(5807):1898–903. doi: 10.1126/science.1132745 17185596

[39] Parra GI, Abente EJ, Sandoval-Jaime C, Sosnovtsev SV, Bok K, Green KY. Multiple antigenic sites are involved in blocking the interaction of GII.4 norovirus capsid with ABH histo-blood group antigens. J Virol. 2012;86(13):7414–26. doi: 10.1128/JVI.06729-11 22532688 PMC3416322

[40] Allen DJ, Noad R, Samuel D, Gray JJ, Roy P, Iturriza-Gómara M. Characterisation of a GII-4 norovirus variant-specific surface-exposed site involved in antibody binding. Virol J. 2009;6:150. doi: 10.1186/1743-422X-6-150 19781066 PMC2762976

[41] Lindesmith LC, Beltramello M, Donaldson EF, Corti D, Swanstrom J, Debbink K, et al. Immunogenetic mechanisms driving norovirus GII.4 antigenic variation. PLoS Pathog. 2012;8(5):e1002705. doi: 10.1371/journal.ppat.1002705 22615565 PMC3355092

[42] Lindesmith LC, Donaldson EF, LoBue AD, Cannon JL, Zheng DP, Vinje J. Mechanisms of GII.4 norovirus persistence in human populations. PLoS Med. 2008;5:0269–90. doi: 10.1371/journal.pmed.0050031 PMC2235898 18271619

[43] Parra GI, Tohma K, Ford-Siltz LA, Pilewski KA, Kendra JA. The saga to monitor and control norovirus: the rise of GII.17. J Gen Virol. 2025;106(6):002118. doi: 10.1099/jgv.0.002118 40476850 PMC12282288

[44] Strother CA, Brewer-Jensen PD, Becker-Dreps S, Zepeda O, May S, Gonzalez F, et al. Infant antibody and B-cell responses following confirmed pediatric GII.17 norovirus infections functionally distinguish GII.17 genetic clusters. Front Immunol. 2023;14:1229724. doi: 10.3389/fimmu.2023.1229724 37662930 PMC10471973

[45] Lindesmith LC, Brewer-Jensen PD, Conrad H, O’Reilly KM, Mallory ML, Kelly D, et al. Emergent variant modeling of the serological repertoire to norovirus in young children. Cell Rep Med. 2023;4(3):100954. doi: 10.1016/j.xcrm.2023.100954 36854303 PMC10040388

[46] Lindesmith LC, Boshier FAT, Brewer-Jensen PD, Roy S, Costantini V, Mallory ML, et al. Immune Imprinting Drives Human Norovirus Potential for Global Spread. mBio. 2022;13(5):e0186122. doi: 10.1128/mbio.01861-22 36102514 PMC9600701

[47] Parra GI, Green KY. Genome of emerging norovirus GII.17, United States, 2014. Emerg Infect Dis. 2015;21:1477–9. doi: 10.3201/eid2108.150652 26196235 PMC4517714

[48] Gao J, Zhang Z, Xue L, Li Y, Cheng T, Meng L, et al. GII.17[P17] and GII.8[P8] noroviruses showed different RdRp activities associated with their epidemic characteristics. J Med Virol. 2022. doi: 10.1002/jmv.28216 36254681

[49] Katoh K, Rozewicki J, Yamada KD. MAFFT online service: multiple sequence alignment, interactive sequence choice and visualization. Brief Bioinform. 2019;20(4):1160–6. doi: 10.1093/bib/bbx108 28968734 PMC6781576

[50] Trifinopoulos J, Nguyen L-T, von Haeseler A, Minh BQ. W-IQ-TREE: a fast online phylogenetic tool for maximum likelihood analysis. Nucleic Acids Res. 2016;44(W1):W232-5. doi: 10.1093/nar/gkw256 27084950 PMC4987875

[51] Reynolds CR, Islam SA, Sternberg MJE. EzMol: A Web Server Wizard for the Rapid Visualization and Image Production of Protein and Nucleic Acid Structures. J Mol Biol. 2018;430(15):2244–8. doi: 10.1016/j.jmb.2018.01.013 29391170 PMC5961936

[52] Tan M, Jiang X. Norovirus gastroenteritis, carbohydrate receptors, and animal models. PLoS Pathog. 2010;6(8):e1000983. doi: 10.1371/journal.ppat.1000983 20865168 PMC2928792

[53] Tin CM, Yuan L, Dexter RJ, Parra GI, Bui T, Green KY, et al. A Luciferase Immunoprecipitation System (LIPS) assay for profiling human norovirus antibodies. J Virol Methods. 2017;248:116–29. doi: 10.1016/j.jviromet.2017.06.017 28673856 PMC5592151

[54] van Loben Sels JM, Meredith LW, Sosnovtsev SV, de Graaf M, Koopmans MPG, Lindesmith LC, et al. A luciferase-based approach for measuring HBGA blockade antibody titers against human norovirus. J Virol Methods. 2021;297:114196. doi: 10.1016/j.jviromet.2021.114196 34019938 PMC9924141

[55] de Graaf M, Bodewes R, van Elk CE, van de Bildt M, Getu S, Aron GI, et al. Norovirus Infection in Harbor Porpoises. Emerg Infect Dis. 2017;23(1):87–91. doi: 10.3201/eid2301.161081 27983498 PMC5176230

[56] van Riel D, Munster VJ, de Wit E, Rimmelzwaan GF, Fouchier RAM, Osterhaus ADME, et al. H5N1 Virus Attachment to Lower Respiratory Tract. Science. 2006;312(5772):399. doi: 10.1126/science.1125548 16556800

